# Seroprevalence of transfusion-transmissible infections among blood donors at National Blood Transfusion Service, Eritrea: a seven-year retrospective study

**DOI:** 10.1186/s12879-018-3174-x

**Published:** 2018-06-07

**Authors:** Nejat Siraj, Oliver Okoth Achila, John Issac, Efrem Menghisteab, Maedn Hailemariam, Semere Hagos, Yosan Gebremeskel, Daniel Tesfamichael

**Affiliations:** 1 Department of Clinical Laboratory Science, Asmara College of Health Sciences, P.O. Box 8566, Asmara, Eritrea; 2 Data Mangement Unit, National Blood Transfusion Service (NBTS), Asmara, Eritrea

**Keywords:** Prevalence, Blood transfusion, Transfusion transmitted infection, National Blood Transfusion Service

## Abstract

**Background:**

Blood transfusion is associated with several risks particularly exposure to blood transfusion-transmissible infections (TTI), including: Hepatitis B virus (HBV), Hepatitis C virus (HCV), Human immunodeficiency virus (HIV) and Syphilis, among others. The threat posed by these blood-borne pathogens is disproportionately high in Sub-Saharan Africa (SSA). This fact underscores the need for continuous surveillance of TTIs in the region. Therefore, the study objectives were to evaluate the prevalence of TTIs and donor characteristics associated with positivity for TTIs at the National Blood Transfusion Center (NBTC) in Asmara, Eritrea.

**Methods:**

A retrospective analysis of blood donors’ records covering the period from January 2010 to December 2016 was undertaken. The records were analyzed to evaluate the annualised cumulative prevalence of TTIs. Chi-square test (χ^2^) or Fisher’s exact test was used to evaluate the relationship between serological positivity and particular donor characteristics. Logistic regression was fitted to identify factors associated with cumulative TTIs positivity. A *P*-value < 0.05 was considered statistically significant.

**Result:**

A total of 60,236 consecutive blood donors were screened between 2010 and 2016. At least 3.6% of donated blood was positive for at least one TTI and 0.1% showed evidence of multiple infections. The sero-prevalence of HBV, HCV, HIV, syphilis and co-infection was 2.0, 0.7, 0.3 and 0.6%, respectively. Sex, type of donor and region were associated with TTI positivity. Except for donation frequency, there was a significant relationship (*P* < 0.005) between HBV, HCV, HIV and syphilis sero-positivity and other donor characteristics evaluated in the study.

**Conclusion:**

The result demonstrates that Eritrea has relatively low TTI prevalence compared to other countries in Sub-Saharan Africa. However, the prevalence, particularly that of HCV, increased significantly in 2016. Enhancing donor screening and additional research utilizing nucleic acid based techniques should therefore be prioritized.

## Background

Blood transfusion is an important therapeutic intervention that has a critical role in patient management [1]*.* Nonetheless, availability of safe blood products is still a significant public health concern in Sub-Saharan Africa (SSA). This phenomenon is driven by a range of overarching factors, including endemicity of infections associated with anemia, high prevalence of sickle cell anemia, blood loss linked to accidents, surgical and/or obstetrical emergencies and malnutrition, among others [2]. Despite the existence of World Health Organization (WHO) approved national hemovigilance protocols in most countries in the region, blood transfusion continues to carry a certain margin of risk for both patients and healthcare workers [3]. Hypersensitivity reactions and direct or residual risk associated with a spectrum of transfusion-transmissible infections (TTI), including Human Immunodeficiency virus (HIV), Hepatitis B virus (HBV), Hepatitis C virus (HCV) and Syphilis-causing *Treponema palladium* (*T. pallidum*), are the major concerns.

In particular, estimates from a previous multicenter study reported that the post-transfusion risk of HIV infection ranges between 1 in 25,600 and 1 in 90,200 [4]. According to some studies, an estimated 25% of blood units donated in Francophone SSA is contaminated with markers of blood-borne pathogens [4]. Others have noted that the widespread use of serological tests and possible misclassification of TTI acquired infections in the region may lead to under-reporting of risks [5, 6]. Overall, the reported proportions of TTIs in SSA are much higher than in industrialised countries [2]. The enhanced odds of acquiring TTIs in the region has been attributed to several challenges, including poor quality test kits and/or unreliable supply of test kits, sub-optimal quality assurance (QA) systems in many centers, shortages of trained laboratory staff, inability to detect recently infected subjects, absence of physical or chemical treatment of blood products [5]. The preponderance of replacement donors (RD) - family members or friends of the patient; as opposed to voluntary non-remunerated blood donors (VNRBD) and the high prevalence of blood-borne infections in the general population in the region has also been linked to the observed TTI associated risk [5].

To reduce TTI-associated risk in SSA, experts have proposed a three-pronged strategy, including improvements in blood donor selection algorithms, more effective TTI detection techniques and chemical and physical treatment of blood products [4]. The latter two strategies are of limited relevance in SSA with the exception of a select number of countries. At present, optimisation of blood donor selection premised on deferral of high-risk prospective donors remains the primary strategy to reduce risk [2]. This strategy emphasizes the need for continuous monitoring of, and reporting on, TTIs. Evaluation of trends in the prevalence of TTIs is not only essential for the assessment of blood safety or the effectiveness of the blood safety strategies; they can also provide an estimate of the epidemiology of sexually-transmitted infections (STI) in the general population [3].

Despite the well recognised importance of studying TTI epidemiology, published information on TTIs burden in SSA is scant [7]. In Eritrea for instance, the last published study on TTIs was conducted in 2009 [8]. This study indicated that the prevalence of TTI markers were 0.18, 2.58, 0.57, and 0.49% for HIV, HBV, HCV and Syphilis, respectively [8]. The scenario may have changed in the interim by virtue of changes in migration flows and urbanization, among others. More importantly, the study had several limitations. For instance, the researchers did not report on the relationship between prevalence of TTIs and associated donor characteristics such as sex, region of residence or age. Therefore, this study not only provides updated information on the prevalence of TTIs and associated factors in Eritrea; it is also more comprehensive given the larger sample size, the expanded number of socio-demographic factors and the longer study duration (7 years).

## Methods

### Study area

The data was extracted from the National Blood Transfusion Service (NBTS) database. NBTS is the only blood bank in Asmara, the capital city of Eritrea. The facility provides TTI-tested blood and blood products for about 20 referral hospitals in the country [8]*.* The center has several departments, sections and sub-sections. It comprises of Donor Clinic section, Laboratory section (sub-sections: TTI, Immunohematology and Component Preparation Sections), Quality Management and Data Management sections.

Donors at the facility are predominantly VNRBD and blood units were collected during blood donation campaigns. All the assays conducted by the TTI sub-section to test for HIV, HBV, HCV and Syphilis were performed in strict compliance with existing national testing policies and guidelines.

### Study design

A retrospective descriptive study of blood donor data recorded at NBTC from January 2010 to December 2016 was undertaken. The information expropriated from the database included: coded donor ID, age, sex, region/zone of residence, type of donation and frequency of blood donation. The outcome variables were HBV, HCV, HIV, Syphilis seropositivity and overall TTI.

### Study population

The study population included all blood donors, 60,236 in total, who donated blood to the NBTS from January 2010–December 2016. Participants were either VNRBD (54272) or RD (5963). Donors were selected based on a pre-set criterion which particularized age (16–65 years), weight (> 50 kg) and medical history as per NBTS protocol.

### Screening methods

The NBTS-TTI screening department used the following assays: HBsAg (SD HBs Ag ELISA 3.0), HCV (SD HCV ELISA 3.0), Syphilis (MEDIFF TPHA) and HIV testing ELISA-1 (Vironostika^R^ HIV-1 Plus O Microelisa System) and ELISA - 2 (AccuDiag™ HIV 1&2 Ag/Ab ELISA ^4th^ Generation) or Murex HIV Ag/Ab Combination (ABBOTT Diagnostics Division).

### Statistical analysis

Data retrieved from the NBTS Delphyn Blood Bank Data Management version 7.6.10.0 database was transferred to Excel spread sheet (Microsoft Corp). The data was subsequently cleaned, recoded and analyzed using IBM SPSS Statistics version 20.0 (Armonk, New York, USA). Prevalence of TTI was expressed as the number of seropositive samples per year. Prevalence of anti-HIV, HBsAg, anti-HCV and anti-TP were expressed in percentages per year for specific donor characteristics. Pearson Chi-squire (χ^2^) test or Fisher’s exact test was used to evaluate the relationship between categorical variants. Chi-square trend test (Linear-by-Linear association) was applied to examine year-by-year variation in trends. Multivariable binary logistic regression was used to evaluate the relationship between TTI (dependent variable) and the influencing factors (sex, donor type and region of residence). A *p* - value < 0.05 was considered statistically significant.

### Ethics approval

Ethical clearance was obtained from Asmara College of Health Science (ACHS) Research Ethical Committee and Eritrean Ministry of Health Research Ethical Committee. Additional approval was obtained from NBTS director. The study utilized previously collected data and no participants were involved at any stage. To guarantee donor confidentiality, donors were anonymized via de-identification (through the use of codes). The process of delinking donor identities and donation units was undertaken exclusively by a data specialist from NBTS. The need to obtain consent from donors was waived by the Eritrean Ministry of Health Research Ethical Committee.

## Result

### Socio-demographic characteristics of the donors

A total of 60,236 blood samples donated to the NBTS from January 2010 to December 2016 were retrieved and analyzed for TTIs. The characteristics of the study population are shown in Table 1. Overall, 66.4% of the donors were males and 33.6% were females. Their ages (in years) ranged from 16 to 54. The age grouping spanning 18–24 years constituted the highest proportion of donors (47.09%) followed by the age group 25–44 years (24.77%). A majority of the study participants were VNRBD (90.1%) with RD accounting for the remaining proportion, 9.9%. Among the donors, 19,816 (32.9%) were first time donors, 17,169 (28.5%) were second time donors and 38.6% had donated blood more than two times. Further, 46.2% of the donors had type O blood group, 25.6% had type B, 23.1% had type A and 5.1% had type AB. In addition to this, 90.6% of the donors were Rh positive while 9.4 were Rh negative.

**Table 1:** Socio-Demographic characteristics of blood donors who donated blood to the NBTS from January 2010 to December 2016

Donor Characteristic		Number (N)	Percentage (%)
Sex	Male	39,978	66.4
	Female	20,258	33.6
Total		60,236	100
Age	16–17	12,666	21.0
	18–24	28,364	47.1
	25–44	14,922	24.8
	45–64	4276	7.1
Total		60,236	100
Region	Maekel	46,420	77.06
	Debub	8860	14.71
	Anseba	2268	3.77
	Northern Red Sea	1435	2.38
	Gash Barka	1002	1.66
	Southern Red Sea	251	0.42
Total		60,236	100
Donor Type	VNRBD	54,264	90.1
	Replacement Donors	5972	9.9
Total		60,236	100
Donation Frequency	First time	19,816	32.9
	Second time	17,169	28.5
	Third time	8668	14.4
	Four and above	14,583	24.2
Total		60,236	100
Blood Type	O	27,841	46.2
	B	15,400	25.6
	A	13,917	23.1
	AB	3078	5.1
Total		60,236	100
RH Type	RH Positive	54,553	90.6
	RH Negative	5683	9.4
Total		60,236	100

*VNRBD* Voluntary non replacement blood donors

### Seroprevalence of TTI among the donors

The TTI results of the 60,236 donations are summarized in Table 2. Among the donors, 3.7% tested positive for at least one TTI. The overall positivity rates of HBV, HCV, HIV, and Syphilis were 2.0, 0.7, 0.3, 0.6 and 0.07%, respectively.

**Table 2:** Positivity rate Transfusion Transmitted Infections (TTI) among the donors by year (January 2010 - Dec 2016)

Year	Donations	Anti-HBsAg+ No (%)	Anti-HCV+ No (%)	Anti-HIV No (%)	Anti -TP+ No (%)	MI No (%)	Total TTI+ No (%)
2010	9977	228 (2.3)	33 (0.3)	37 (0.4)	67 (0.7)	5 (0.05)	370 (3.7)
2011	10,080	178 (1.8)	54 (0.5)	29 (0.3)	91 (0.9)	8 (0.08)	360 (3.6)
2012	9219	156 (1.7)	31 (0.3)	33 (0.4)	70 (0.8)	5 (0.05)	295 (3.2)
2013	8661	181 (2.1)	63 (0.7)	26 (0.3)	35(0.4)	8 (0.09)	313 (3.6)
2014	8051	177 (2.2)	64 (0.8)	21 (0.3)	36 (0.4)	4 (0.05)	302 (3.8)
2015	7006	159 (2.3)	42 (0.6)	16 (0.2)	21 (0.3)	6 (0.09)	244 (3.5)
2016	7242	124 (1.7)	155 (2.1)	20 (0.3)	31 (0.4)	6 (0.08)	336 (4.6)
*P* - value		0.004	0.000	0.613	0.000	0.864	0.000
Total	60,236	1203 (2.0)	442 (0.7)	182 (0.3)	351(0.6)	42(0.07)	2220 (3.7)

*P*-value, Pearson Chi-square test for statistical difference in the distribution within each group, *MI* Multiple Infections

The most prevalent type of TTI throughout the 7 years was HBV. The proportion of donors infected with at least one TTI was 3.6 and 0.1% had multiple infections (Maximum = 3). The frequency of TTIs was comparatively higher in 2016 (4.87%) and lowest in 2010 (2.8%). The concurrent infection with the highest frequency was HBV-Syphilis. Over the 7-year period, Chi-squire trend test demonstrated a significant increase (*p* < 0.00) in TTI trends from 2013 (3.2%) to 2016 (4.6%).

Seropositivity of TTI within specific socio-demographic categories, including sex, age and region of residence were evaluated. Table 3. According to our data, male donors had a significantly higher frequency of TTIs compared to female donors (4.0% versus 2.97%, *p* < 0.00). In particular, male donors had comparatively higher frequencies of HBV (2.2% versus 1.5%, *p* < 0.00), HCV (0.8% versus 0.6%, *p* < 0.026) and syphilis (0.7% versus 0.4%, *p* < 0.00). In contrast, female donors had a marginally higher frequency of HIV compared to male donors (0.4% versus 0.3%, *p* < 0.005). Mixed infections (MI) were only observed in male donors (0.09%) (*p* < 0.05).

**Table 3:** Positivity rate of transfusion – transmissible infections by sex, age group and region of residence of the donors

Donor Characteristic	No of Donors (N)	Positive No (%)	HBsAg+ No (%)	Anti-HCV+ No (%)	Anti - HIV+ No (%)	Anti -TP+ No (%)	MI No (%)
Sex							
Male	39,978	1619 (4.0)	892 (2.2)	313 (0.8)	104 (0.26)	275 (0.7)	35 (0.09)
Female	20,258	601 (2.97)	311 (1.5)	129 (0.6)	78 (0.39)	76 (0.4)	7 (0.0)
*P* -value		0.000*	0.000*	0.026*	0.005*	0.000*	0.015*
Age Group							
16–17	12,666	566 (4.4)	331 (2.6)	115 (0.9)	53 (0.4)	61 (0.5)	6 (0.0)
18–24	28,364	817 (2.9)	467 (1.6)	181 (0.6)	57 (0.2)	94 (0.3)	18 (0.1)
25–44	14,922	614 (4.1)	322 (2.2)	114 (0.8)	54 (0.4)	112 (0.8)	12 (0.1)
45–64	4276	223 (5.1)	83 (1.9)	32 (0.7)	18 (0.4)	84 (2.0)	6 (0.1)
*P* -value		0.000	0.000	0.000	0.001	0.000	0.357
Region of Residence							
Maekel	46,420	1449 (3.1)	731 (1.6)	291 (0.6)	145 (0.3)	258 (0.6)	24 (0.1)
Debub	8860	455 (5.1)	285 (3.2)	87 (1.0)	26 (0.3)	49 (0.6)	8 (0.1)
Gash-Barka	1002	95 (9.5)	51 (5.1)	14 (1.4)	1 (0.1)	24 (2.4)	5 (0.5)
Anseba	2268	120 (5.3)	71 (3.1)	35 (1.5)	5 (0.2)	6 (0.3)	3 (0.1)
North Red Sea	1436	89 (6.2)	57 (4.0)	13 (0.9)	4 (0.3)	13 (0.9)	2 (0.1)
South Red Sea	251	12 (4.8)	8 (3.2)	2 (0.8)	1 (0.4)	1 (0.4)	0 (0.0)
*P* -value		0.000	0.000	0.000	0.828	0.000	0.000

*P*-value, Pearson Chi-square test and Fishers exact test (*) for statistical difference in the distribution within each group, *MI* Multiple Infections

A statistically significant association was also observed between age categories and TTI seropositivity (*p* < 0.00). The data shows that the frequency of TTI among donors within 45–64, 25–44, 18–24 and 16–17 years of age were 5.1, 4.1, 2.9 and 4.4%, respectively. In particular, TTI positivity was higher in donors above 45 years of age (5.1%) and donors below 17 years of age (4.4%). Additionally, a significant association was observed between age categories and evaluated TTIs (*p* < 0.05). Markers for HBV and HCV were more prevalent in donors below 17 years (2.6%) of age and lowest in donors between 18 and 24 years of age (1.6%). In contrast, the frequency of anti - *T. pallidum* antibodies was higher in donors above 45 years (2%) and lowest in donors between 18 and 24 years (0.3%).

Regionally, donors from Gash-Barka region had the highest rate of infection (9.5%) and donors from Maekel zone had the lowest TTI cases (3.1%), *p* < 0.00. Except for HIV, regional variation in the frequency of specific TTIs was observed (*p* < 0.00). Donors from Gash-Barka had the highest frequency of HBV (5.1%), anti-*T. pallidum* markers (2.4%) and co-infections (0.5%) compared to the other regions. Additionally, frequency of HCV markers was significantly higher in Anseba (1.5%), nearly as high in Gash-Barka (1.4%) and lowest in Maekel (0.6%), *p* < 0.000.

In this study, RD donors had a higher frequency of TTI compared to VNRBD (6.9% versus 3.4%, *p* < 0.000). In particular, the prevalence of TTI markers was significantly higher in RD compared to VNRBD: (3.6% versus 1.9%, *p* < 0.000 for HBV), (1.0% versus 0.7%, *p* < 0.006 for HCV), (0.6% versus 0.3%, *p* < 0.000 for HIV) and (1.7% versus 0.5%, *p* < 0.000 for HIV. In addition, the frequency of coïnfections was significantly higher in RD (0.2%), *p* < 0.007. Among all donors, the prevalence of TTIs was 10.8% for first time donors, 0.16% for second time donors, 0.53% third time donors and 0.021% for donors who had donated blood more than three times (*p* < 0.000). Table 4.

**Table 4:** Positivity rate of transfusion – transmissible infections (TTI) by donor type and donation frequency of the donors

Variables	No of Donor	Positive No (%)	HBsAg+ No (%)	Anti-HCV+ No (%)	Anti - HIV+ No (%)	Anti -TP+ No (%)	MI No (%)
Donor Type							
VNRBD	54,264	1808 (3.3)	1031 (1.9)	382 (0.7)	146 (0.3)	249 (0.5)	32 (0.1)
Replacement	5972	412 (6.9)	214 (3.6)	60 (1.0)	36 (0.6)	102 (1.7)	10 (0.2)
***P*** **-value**		0.000*	0.000*	0.006*	0.000*	0.000*	0.007*
Donation Frequency							
First time	19,816	2142 (10.8)	1165 (5.8)	426 (2.2)	172 (0.9)	339 (1.7)	40 (0.2)
Second time	17,169	29 (0.16)	12 (0.06)	8 (0.05)	3 (0.05)	6 (0.03)	0 (0.0)
Third time	8668	46 (0.53)	25 (0.29)	8 (0.09)	6 (0.07)	5 (0.06)	2 (0.02)
Four & above	14,583	3 (0.021)	1 (0.007)	0 (0.0)	1 (0.007)	1 (0.007)	0 (0.0)
*P* - value		0.000	0.048	0.572	0.240	0.561	0.548

*P*-value, Pearson Chi-square test and Fishers exact test (*) for statistical difference in the distribution within each group, *MI* Multiple Infections. *VNRBD* Voluntary non-replacement blood donors

Multivariate logistic regression analysis was undertaken to determine the donor characteristics and association to TTI. In this analysis, male donors (AOR = 0.741; 95% CI: 0.672, 0.816) were less likely to be seropositive compared to female donors. Further, VNRBD (AOR = 2.103 95% CI: 1.880–2.351) were more likely to be seropositive for TTIs compared to RD. Donors from Gash-Barka (AOR = 0.475, 95% CI, 0.256, 0.882) less likely to be seropositive for TTI. Table 5.

**Table 5:** Multivariate Logistic regression model for demographic predictors of TTI

Catégories	*P*- values	AOR (95% CI)
Sex		
Female^a^		1
Male	0.000	0.741 (0.672–0.816)
Type of Donor		
Replacement Donor^a^		1
VNRBD	0.000	2.103 (1.880–2.351)
Region of Residence		
Southern Red Sea^a^		1
Maekel	0.192	1.475 (0.823–2.645)
Debub	0.519	0.824 (0.457–1.485)
Gash-Barka	0.018	0.475 (0.256–0.882)
Anseba	0.574	0.840 (0.456–1.545)
Northern Red Sea	0.293	0.717 (0.386–1.333)

^a^- indicates reference. *AOR* Adjusted Odds Ratio, *CI* Confidence Interval, *VNRBD* Voluntary non-replacement blood donors

## Discussion

Eritrea faces critical challenges in blood safety and availability. The high frequency of blood borne viruses and other infectious diseases, including HIV, HBV, HCV and syphilis continue to be a major concern. Although epidemiological reports published in recent years indicate that significant progress has been made in countries where WHO national hemovigilance protocols have been implemented; risk of TTIs is still an issue in Eritrea. Therefore, monitoring prevalence trends of a spectrum of TTIs in donor population remains a valuable index for evaluating the effectives of existing intervention strategies.

In this study, we established that the overall cumulative frequency of TTI markers in donated blood was 3.7%. The finding compares favorably to a previous study in Eritrea which reported a prevalence of (3.8%) [8]. However, it is lower than the findings from other multicenter and local studies in SSA. For instance, previous studies in Ethiopia [3], Mozambique [9], Equatorial New Guinea [10] and Burkina Faso [11] reported a seroprevalence of 6.55, 18.7, 37.39 and 24%, respectively. In contrast, lower prevalence was reported in earlier studies undertaken in Namibia (1.4%) [12] Iran (0.254%) [13] and China (2.67%) [14].

Several issues should be noted in the reported results. Of note is the fact the frequency of TTI markers reported in this study are comparatively low. Nevertheless, Chi squire (χ^2^) trend test demonstrated an increasing trend. However, it should be emphasised that the sharp uptick in the frequency of HCV in 2016 account for much of the observed trend. Interestingly, significant reduction in the frequency of syphilis and anti-HBV makers was also noted. The lower frequency of TTIs may be attributed to several factors, including the relatively low prevalence of blood-borne pathogens such as HIV in the general population [15] and more effective pre-donation screening designed to exclude high risk donors and the higher frequency of VNRBD, among others. Another important finding is that the frequency of TTI in RD was comparatively higher (6.9% versus 3.3% for VNRBD). A similar finding was reported by researchers working elsewhere [16–18]*.* In general, RD donors are regarded as a high risk group. This adequately corroborated presumption has been linked to a variety of factors including the possibility that family members and friends are more likely to provide false information on issues that might invite further inquiry thereby limiting the level of protection provided by risk-screening questionnaires [19]. In addition, family members may be at a higher risk for some infection due to a shared exposure to specific predisposing factors or environment, for example, HBV.

Furthermore, the frequency of TTI markers in this study was significantly associated with sex with a higher prevalence reported in male donors. A similar association was reported in a study conducted at the Jijiga Blood Bank, Eastern Ethiopia (11.6% males versus 3.8% females) [16]*.* The higher proportion of male donors presenting with TTI markers may be attributed to behavioral, religious and socio-cultural drivers of high-risk sexual behavior that are typically found in conservative societies. Another plausible explanation of this disproportionate male to female TTI positivity ratio may be related to the fact that females are better diagnosed in Eritrea due to pre-natal care. Thus, a higher proportion of female blood donors may be aware of their seronegative status, at least for some TTIs. A similar suggestion was previously proposed to explain comparable results in a study conducted in Brazil [19]. The frequency of TTI markers was also found to be higher in RD compared to VNRBDs in this study. A higher frequency of TTI was also observed in first-time donors. The results provide additional support to the general notion, widespread in blood transfusion literature, that first-time donors constitute a high risk group [2]. This position is premised on the suggestion that these donors have ambiguous motive to donate and have had no previous pre-donation screening for TTIs. Moreover, the possibility of accessing screening for particular TTIs (HIV for instance) may incentivise some individuals to donate blood. Others may be incentivised by the possibility of financial compensation.

The prevalence of TTI markers in donors in the evaluated age categories was also different. This outcome is in line with findings from others studies [14]. The frequency of TTI markers was substantially higher in donors above 45 years and in those below 17 years. Donors within the 18–24 year age grouping had the lowest TTI prevalence. The explanation for the observed pattern may be associated with behavioral characteristics unique to these age categories. Conversely, the finding may indicate that the intervention measures implemented in the country may have disproportionate impact on the disparate age groupings. Regional disparity in TTI prevalence was also observed with donors from Gash-Barka recording generally higher prevalence. This may be ascribed to cultural differences and regional variations in general levels of education, among others.

Although the frequency of TTI reported in this study is nearly similar to that of a previous local study, significant differences pertaining to seroprevalence and trends of particular TTIs was observed. The most dominant type of TTI over the study duration was HBV (2.0%). The reported prevalence was lower when compared to results obtained from similar studies conducted in the region [1, 7, 20]. Further, the high prevalence of HBV among blood donors in SSA has been attributed to the high prevalence [8%] and endemicity of the virus in the region [2]. A point reflected in estimated transfusion risk models which place HBV above HIV in Africa [2]. These results point to the possibility that the prevalence of HBV in the general population maybe comparatively high. Equally notably is the fact that prevalence of HBV markers exhibited a statistically significant association with sex, age categories, regions and donor type. Behavioural, cultural and socio-economic differences associated with membership to these categories may explain the observed variation. In particular, the high seroprevalence of HBV markers (and HCV) in donors below 17 years of age may be linked to the fact that most young people acquire piercings or tattoos at this age - This hypothesis is highly speculative and warrants further investigation.

The overall prevalence of HCV was 0.7%. This was slightly higher than findings from previously studies from Southern Africa [9, 12]. Although this value compares favourably to findings from a study in North West Ethiopia [3], the prevalence was lower when compared to studies conducted in Egypt (4.3%) [21]*,* Tanzania 1.5% [22], Ethiopia [1] and Sudan 3.4% [23]. This result corroborates the assertion that HCV poses less risk to blood transfusion in Southern parts of Africa by virtue of low prevalence [2]. However, it is important to note that the presence of anti-HCV antibodies among apparently healthy blood donors in Eritrea confirms the presence of HCV in the country. In addition, our data also demonstrated that there has been an upward trend in HCV prevalence over the years – the sharp uptick in 2016 is particularly noteworthy. Although this finding should raise concern, it should be viewed in light of previous reports which noted that HCV antibody tests tend to have high false positive misclassification in African populations [24, 25]. The high frequency of false-positive antibodies (FPA) for HCV has been associated with infectious agents such as malaria, *Schistosoma mansoni*, Syphilis, HIV, malnutrition or other chronic infections [24]. Therefore, it’s our opinion that in the absence of confirmatory nucleic acid based tests (NAT) for HCV; categorical estimates of the frequency of HCV among blood donors in this population may be elusive.

According to this study, the prevalence of HIV among blood donors was 0.7%. The proportion is considerably lower compared to other studies conducted elsewhere in Africa [1, 7, 10, 21, 26]. However, it was higher than the results obtained in Libya (0.014%) [27] and Egypt (0.00%) [28]. The observed prevalence shows consistency with reported HIV seroprevalence in Eritrea [29]. Compared to a previous study, disproportionate HIV prevalence was observed between RD and VNRBD [8]. An association between age grouping and sex was also observed. Higher burden of infection occurred in females and in individuals below 17 years and above 25 years. An explanation for the relatively lower proportion of HIV seroprevalence in males and in donors between 18 and 24 years of age is not apparent. This situation is particularly remarkable given the fact that this is the only infection in which women exhibit a higher burden of disease compared to males. Moreover, Syphilis, a known predisposing factor for HIV, was significantly higher in males.

Treponemal positivity prevalence of 0.6% was also reported in this study. The observed value is lower compared to studies conducted in the region [1, 10, 30]. The prevalence of syphilis was highest in Ghash-Barka and among individuals aged between 45 and 64 years of age. This finding provides additional support for a recent review which concluded that higher prevalence of *T. pallidum* markers is normally found in elderly donors and in donors with low level of education [31]. Equally notable is the fact that Gash-Barka has a significant proportion of pastoralist communities who tend to have low level of education and are hard to reach with health intervention measures.

### Limitation of the study

This study was a retrospective in design. Therefore, we were not able to control, or provide information on, a range of factors which may have had a bearing of the results. For instance, the range of risk factors evaluated in this study was limiting. Indeed, a screening questionnaire customised to the study environment may have provided more insight on the opportunities for optimization of donor selection and testing algorithm from a local standpoint. Another weakness may be associated with intrinsic weakness of the diagnostic test used in this study – use of serological as opposed to nucleic acid based techniques. Therefore, the results reported in this study may underestimate (presence of a window period) or overestimate (high rate of false positivity HCV) the frequency of TTIs among donors in this population.

## Conclusion

The overall prevalence of TTIs in Eritrea is comparatively low. This situation may be attributed to several factors, including an effective system for donor recruitment, selection and screening, among others. However, the study demonstrated the fact that the risk of TTIs is still substantial, a problem which is compounded by indicators; reported in this study, which appear to suggest that the frequency of specific TTIs such as HCV have increased marginally over time. According to the study, HBV followed by HCV, had the highest frequency in Eritrea. The prevalence of the TTIs evaluated in this study was associated with sex, region/zone of residence, type of donor and frequency of donation. In particular, the prevalence of TTI was high among male, RD and donors from Gash Barka region. The relatively high prevalence of TTIs (HBV and HCV, in particular), in donors below 17 years of age is also noteworthy. Therefore, there is an urgent need to investigate the epidemiology of TTIs in Eritrea. This can be achieved through continuous monitoring of the prevalence of TTI-markers in the population via the utilization of contextualised screening questionnaires and the use of NAT methods.

## Abbreviations

ACHS: Asmara College of Health Science
AOR: Adjusted odds ratio
CI: Confidence interval
ELISA: Enzyme linked immunosorbent assay
FPA: False-positive antibodies
HBV: Hepatitis B virus
HCV: Hepatitis C virus
HIV: Human immunodeficiency virus
NBTS: National blood transfusion service
NRS: Northern red sea region
RD: Replacement donors
SPSS: Statistical package for social services
SRS: Southern red sea region
TPHA: *Treponema Pallidum* Hemoagglutination Assay
TTI: Transfusion transmitted infection
VNRBD: Voluntary non-replacement blood donors
